# Enteric Bacterial Metabolites Propionic and Butyric Acid Modulate Gene Expression, Including CREB-Dependent Catecholaminergic Neurotransmission, in PC12 Cells - Possible Relevance to Autism Spectrum Disorders

**DOI:** 10.1371/journal.pone.0103740

**Published:** 2014-08-29

**Authors:** Bistra B. Nankova, Raj Agarwal, Derrick F. MacFabe, Edmund F. La Gamma

**Affiliations:** 1 New York Medical College, Department of Pediatrics/Maria Fareri Children's Hospital, Valhalla, New York, United States of America; 2 The Kilee Patchell-Evans Autism Research Group, Departments of Psychology (Neuroscience) and Psychiatry, Division of Developmental Disabilities, The University of Western Ontario, London, Ontario, Canada; North Carolina State University, United States of America

## Abstract

Alterations in gut microbiome composition have an emerging role in health and disease including brain function and behavior. Short chain fatty acids (SCFA) like propionic (PPA), and butyric acid (BA), which are present in diet and are fermentation products of many gastrointestinal bacteria, are showing increasing importance in host health, but also may be environmental contributors in neurodevelopmental disorders including autism spectrum disorders (ASD). Further to this we have shown SCFA administration to rodents over a variety of routes (intracerebroventricular, subcutaneous, intraperitoneal) or developmental time periods can elicit behavioral, electrophysiological, neuropathological and biochemical effects consistent with findings in ASD patients. SCFA are capable of altering host gene expression, partly due to their histone deacetylase inhibitor activity. We have previously shown BA can regulate tyrosine hydroxylase (TH) mRNA levels in a PC12 cell model. Since monoamine concentration is known to be elevated in the brain and blood of ASD patients and in many ASD animal models, we hypothesized that SCFA may directly influence brain monoaminergic pathways. When PC12 cells were transiently transfected with plasmids having a luciferase reporter gene under the control of the TH promoter, PPA was found to induce reporter gene activity over a wide concentration range. CREB transcription factor(s) was necessary for the transcriptional activation of TH gene by PPA. At lower concentrations PPA also caused accumulation of TH mRNA and protein, indicative of increased cell capacity to produce catecholamines. PPA and BA induced broad alterations in gene expression including neurotransmitter systems, neuronal cell adhesion molecules, inflammation, oxidative stress, lipid metabolism and mitochondrial function, all of which have been implicated in ASD. In conclusion, our data are consistent with a molecular mechanism through which gut related environmental signals such as increased levels of SCFA's can epigenetically modulate cell function further supporting their role as environmental contributors to ASD.

## Introduction

The gut microbiota - the diverse range of symbiotic gut bacteria and other microorganisms is involved in the regulation of multiple host metabolic pathways in both health and disease [1], [2]. There is increasing evidence this microbial ecosystem, which outnumber host cells 100 to one, act as a functional “organ”, playing a major regulatory role in gut–brain communication, brain function and even behavior [3], [4], [5], [6], [7]. The mutually beneficial relationship between the host and gut microorganisms arises in part from SCFAs which are produced from bacterial fermentation of some proteins and dietary fiber, the most abundant of which are acetate, BA and PPA [8]. These SCFA serve local functions in phenotypic reprogramming of colonic epithelial cells, as the principal energy substrate for epithelial cells, as tumor suppressor agents, in apoptotic cell death, and in gene regulation of anti-inflammatory processes both *in vitro* and *in vivo* [9], [10], [11], [12], [13], [14], [15].

The majority of SCFA are absorbed from the gut, transported via the portal vein and metabolized in the liver before reaching the systemic circulation. Thus, hepatic clearance was thought to impede achieving levels sufficiently high to affect systemic functions or metabolic and regulatory pathways. However, the distal colon bypasses the portal circulation enabling systemic access. Despite the difficulty in their measurement due to intracellular concentration and rapid metabolism, there is growing evidence that the potential systemic effects of SCFA (especially PPA and BA) on physiology and pathology may have been underestimated (rev. in [8], [3]).

SCFA have a number of direct effects on gastrointestinal physiology. Along with acetate and butyrate, PPA is known to reduce gastric motility and increase the frequency of contractions, presumably via a reflex that involves direct contact of these short chain fatty acids with the terminal ileum [16]. In addition, PPA increases contraction of colonic smooth muscle [17], dilates colonic arteries [18], activates mast cells [19] and increases the release of serotonin from gut enterochromaffin cells [20], [21]. In spite of the multiple beneficial effects of SCFA on host gastrointestinal activity, excessive quantities of PPA have been reported in gingival inflammation [22], acne [23], irritable bowel syndrome [24] and in the neurometabolic disorder propionic acidemia [25]. In this heterogeneous inborn error of metabolism, which may be underreported [26], accumulation of PPA is associated with developmental delay, seizure and extrapyramidal findings, often accompanied by gastrointestinal symptoms [25], [27], confirming that a homeostatic balance of SCFA may be necessary. Furthermore PPA and related SCFA have broad effects on nervous system physiology, including activation of specific G protein coupled receptors (GPCR), neurotransmitter synthesis and release, intracellular pH/calcium gating, mitochondrial function, lipid metabolism, immune function, gap junction gating and gene expression (see [3]).

Recent evidence suggests potential, but unproven, links between dietary, metabolic, immune, infective, and gastrointestinal factors and ASDs. Although inheritable factors, mostly implicated in synaptic transmission, have been traditionally studied in ASD [28], the fact that 1) known genetic factors thus far account for only 10–20% of cases, 2) there is less than 100% concordance in identical twins, and 3) there is a growing prevalence in the condition, collectively suggest an important role for environmental factors which act on the underlying genetic sensitivities [29]. In particular hospitalization, early infections and associated antibiotic exposure [30], which are risk factors for ASD, may alter the developing gut microbiome [2]. Increased mean levels of PPA in stool of ASD children have been shown [31] although contrasting findings were also reported in ASD participants with higher usage of probiotics and fish oil consumption [32]. Given that PPA is a key fermentation product of ASD-associated antibiotic resistant bacteria (*Clostridia*, *Bacteriodetes*, *Desulfovibrio*) [33] and modulates many ASD related biochemical processes, we have proposed that SCFAs represent a group of host microbiome metabolites that are plausibly linked to ASDs and can induce widespread effects on gut, brain, and behavior [3], [4].

Further to this, we [34], [35], [36], [37], [38], [39], [40] and others [41], [27], [42], [43], [44] have shown that short-term central nervous system (intracerebroventricular) and peripheral administration (intraperitoneal, subcutaneous or oral gavage) of PPA, and to a lesser extent, other SCFAs at various developmental time periods in rodents induce ASD-like abnormal motor movements, repetitive interests, electrographic changes, cognitive deficits, perseveration, and impaired social interactions. The brain tissue of PPA-treated rats also shows a number of ASD-linked neurochemical changes, including neurotransmitter alterations, innate neuroinflammation, increased oxidative stress, redox changes, glutathione depletion, and altered phospholipid/acylcarnitine profiles. This novel model also has shown predictive value for potential metabolic biomarkers in a large cohort of ASD patients [45].

One potential key mechanism where the metabolic products of an altered gut microbiome may contribute to ASD pathophysiology is via the alteration of gene expression associated with ASD mutations or ASD implicated genetic pathways [3], [46], [7]. Interestingly, PPA, related SCFA and their derivatives are known modulators of gene expression via their histone deacetylase inhibitor (HDACI) activity [47], [48], [49], [50], [51], [52], [53].

The rat pheochromocytoma (PC12) cell line is an extensively used *in vitro* cell system to examine molecular biological processes in neurobiology [54]. We have used this PC12 line to examine the effects of SCFA, principally BA, and their derivatives on gene expression [55], [56], [57], [58]. Of note, our results underscored at least 3 major mechanisms by which BA can regulate TH gene expression: i) modulation of gene transcription by chromatin remodeling, ii) b**y** activation of transcription factors via different signaling cascades (including Ca2+/cAMP mediated activation of CREB [59]) and involving induction of transcription via an upstream 5′ regulatory element (BRE; GCCTGG at −509 to −504 of the rat TH promoter [60] or iii) by affecting the stability of TH mRNA (e.g. via a butyrate response factor (BRF) acting at a 3′ untranslated AU-rich regions of mammalian mRNAs [61], [62], [63], [64]). Many of the affected genomic pathways are involved in catecholamine synthesis, which have been implicated in ASD [65], [66]. Moreover, CREB, a key factor in neurodevelopment, learning and memory [67], is a key determinant of catecholamine synthesis in PC12 cells, and shows increased CREB immunoreactivity in brains of PPA treated rats (our animal model of ASD, [35]). Furthermore, the anti-seizure/mood stabilizing drug valproic acid, a known prenatal risk factor for ASD, which produces an acceptable animal model for the condition, is structurally and pharmacologically similar to PPA, including HDACI properties [68], [69], [70] and produces similar effects as BA in PC12 cells [58].

Since most research on the effects of SCFA on gene expression is limited to BA and not PPA, in the present study we used rat PC12 cells as an *in vitro* system to extend our observations on the epigenetic effects of SCFA. Microarray technology was used to compare global changes in gene expression profiles following exposure to the structurally related SCFA PPA and BA. Furthermore, we sought to determine if the expression of these PPA dependent genes was related to canonical biological pathways implicated in ASD.

## Methods

### Cells/Transfection

PC12 cells ([71], rat pheochromocytoma of sympathoadrenal origin) were used as a model to delineate the molecular effects of propionate. They were cultured in DMEM media supplemented with 10% horse serum, 5% fetal bovine serum and antibiotics in a humidified atmosphere and 10% CO_2_ as described earlier [64]. Sodium propionate (Sigma, St. Louis, MO) was added at the indicated concentrations at nearly 50% confluence. To evaluate the effect of PPA on TH gene transcription we used a transient transfection approach. Briefly, plasmid constructs with a rat TH promoter (−773/+27 bp, [72] driving the expression of firefly luciferase reporter gene were electroporated into PC12 cells at 300 V and 500 µF with a total of 50 µg/plate of plasmid DNA (10^6^–10^7^ cells). Cells treated with SCFA continued to receive media supplemented with SCFA after electroporation. After incubation for an additional 24 hours, cells were harvested and crude cell lysates were prepared. Protein concentrations in total cell lysates were determined [73] and luciferase reporter gene activities were measured by luminometry as recommended by Promega (Madison, WI). All averages were made from at least six independent assays. The results were given as relative light units per second per microgram of total protein (RLU/s/µg). The luciferase activities were expressed as fold stimulation relative to cells co-transfected with the same constructs but not treated with SCFA. The values shown are means ± SEM from three experiments.

Wild type or dominant negative CREB expression vectors were purchased from BD Biosciences (Palo Alto, CA). All plasmid DNA constructs were purified by chromatography on Qiagen columns (Santa Clarita, CA).

### Isolation of RNA and Northern blot analysis

To compare the effects of structurally related SCFA on endogenous TH gene expression, PC12 cells were treated with low concentrations of SCFA (PPA, BA, and VPA) or with vehicle for 48 hours. Total RNA was isolated from individual petri dishes using RNazol according to the manufacturer's protocol (Tel-Test, Inc., Friends-Wood, TX). Northern blot analysis was performed as described previously [64]. After transfer to Gene Screen Plus membranes, the filters were hybridized consecutively to labeled rat cDNA probe for TH and a probe for 18S ribosomal RNA. Filters were washed under proper stringency and exposed for autoradiography, using Kodak Bio Max film (Rochester, NY). The autoradiographs were scanned and quantified by Bio-Rad Quantity One software and the abundance of TH mRNA was expressed relative to 18S ribosomal RNA levels. The results were presented as fold change compared with the control (vehicle treated) group on the same Northern blot.

### Microarrays

PC12 cells were treated with 1 mM PPA, 10 mM PPA, 1 mM BA, 6 mM BA or vehicle for 48 hrs. Total RNA samples from each experimental group were pooled together and after DNase treatment purified using RNeasy Mini Kit (Qiagen Sciences, MD) as recommended by Affymetrix. The cDNA synthesis and microarray analyses were performed at Keck Affymetrix GeneChip Resource at Yale, New Haven, CT (NIH Neuroscience Microarray Consortium). The chip data was analyzed using Affymetrix GeneChip Operating Software (GCOS) version 1.2.1. Genes showing altered expression with fold change values of ≥2.0 were exported for functional annotation to MetaCore by GeneGO, Inc. The expression levels of selected genes (TH, DBH, NPY, ChrA, PENK, and GTPCH) were validated through Northern blot analyses of the same RNA samples (Table 1). Raw and quantile-normalized microarray data and an associated project metadata file are available through the NCBI-GEO repository (GSE56516).

**Table 1:** Effect of PPA and BA on neurotransmitter related gene expression in PC12 cells.

Gene/fold change	1 mM BA	6 mM BA	1 mM PPA	10 mM PPA
**TH**	↑2.8±0.21	↓6.4±0.12	↑2.17±0.21	↓2.2±0.35
**DBH**	↑1.4±0.17	↓14.5±0.89	↑1.2±0.23	↓1.8±0.13
**NPY**	↑2.78±0.35	↑5.2±0.64	1.02±0.28	↑ 1.3±0.35
**ChrA**	↑1.8±0.15	↓3.0±0.48	↑1.15±0.11	↓0.85±0.12
**PENK**	↑4.65±0.39	↑2.46±0.22	↑2.7±0.25	↑1.95±0.25
**GTPCH**	1.02±0.09	0.98±0.15	↑1.45±0.23	↑2.67±0.27

PC12 cells were treated with 1 mM PPA, 10 mM PPA, 1 mM BA, 6 mM BA or vehicle for 48 hrs. Total RNA samples were isolated from individual petri dishes (n>6 per experimental group). Northern blot analysis was performed as described in Methods section, using probes specific for rat genes, encoding tyrosine hydroxylase (TH), dopamine beta-hydroxylase (DBH), neuropeptide Y (NPY), Chromogranin A (ChrA), pre-proenkephalin (PENK) and GTP cyclohydrolase (GTPCH). The results are presented as mean ± SEM value relative to the mRNA levels in control, vehicle treated cells. 18S rRNA was used as a house keeping gene for normalization of data.

### Western blot analysis

Total protein extracts were prepared from each petri dish of control (vehicle treated) and SCFA- treated PC12 cells for the indicated periods of time (see the figure legend). Proteins were separated on 10% SDS-PAGE, electroblotted onto a nitrocellulose membrane (BioRad; Hercules, CA) and incubated with TH antibody (1: 4000, Imgenex, San Diego, CA) overnight. After incubation with secondary antibody (Goat Anti-Rabbit IgG, from Pierce, Rockford IL; diluted 1: 40000) the immune reaction was visualized by enhanced chemiluminescent substrate from Pierce, utilizing a horseradish peroxidase label and Kodak XAR-5 film, as described by the manufacturer. To confirm equal loading, blots were re-probed with primary antibody for the house keeping protein β-actin (Monoclonal Anti-β-Actin antibody, Sigma, St. Louis, Mo). The blots were exposed to autoradiography and the x-ray films were scanned and quantified with Bio-Rad Quantity One software. The ratio TH/β-actin immunoreactivity were calculated for each sample and the results were presented as fold induction compared to the corresponding control group on the same Western blot.

### Statistical analysis

Data were expressed as mean ± SEM and normalized to the values in the control, taken as 1.0. Differences between the experimental groups from at least three independent experiments were evaluated by performing ANOVA followed by the Fisher's least significant difference test for experiments with more than two groups. A level of p≤0.05 was accepted as statistically significant.

## Results

### 1. PPA induces TH gene transcription in a CREB-dependant fashion

**Figure 1:**
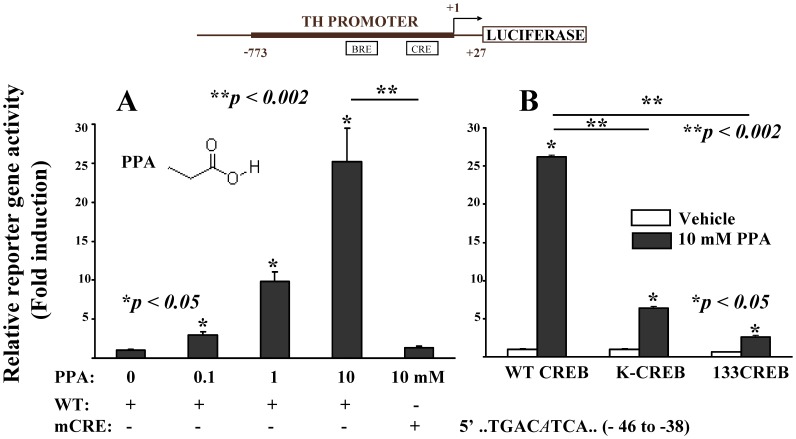
PPA activates the transcription of TH gene in CREB-dependant manner. Plasmid constructs with wild type (WT) rat TH promoter (−773/+27 bp) driving the expression of luciferase reporter gene or having a G-to A transition mutation in the CRE promoter element (mCRE) were electroporated in PC12 cells pre-treated for one day with increasing concentrations of PPA (0.1 to 10 mM, Fig. 1A)). The same doses of PPA were given immediately after electroporation for additional 24 hours, when changes in reporter gene activity were determined. In the second set of experiments (Figure 1B), PC12 cells were co-transfected with combination of wt TH promoter construct and plasmids expressing wild type or dominant negative CREB mutants (binding-defective K-CREB or phosphorylation-defective 133CREB). To half of the cultures, 10 mM PPA was given 1 day before and immediately after electroporation for additional 24 h (black bars). Controls received vehicle 24 hrs before and immediately after electroporation (open bars). The values shown are means ± SEM from three experiments. The luciferase activities (RLU/s/µg protein) are expressed as fold stimulation relative to control cells co-transfected with the same plasmids but not treated with PPA. *P<0.05 (PPA vs. vehicle treated; **P<0.002 (wt vs. mutant constructs).

We have shown before [63], [64], [60], [58] that HDACI like BA and VPA can also activate transcription of the TH gene via upstream regulatory elements in PC12 cells. To test whether PPA has similar effects we performed transient transfection experiments following the same protocol. Briefly, plasmids carrying the luciferase reporter gene under the control of the rat TH promoter (−773/+27 bp) were electroporated into PC12 cells pre-treated for one day with vehicle or with SCFA. Reporter gene activity was measured in control and SCFA-treated culture samples 24 hours after transfection. PPA increased luciferase activity in a concentration-dependent manner consistent with activation of TH promoter (Figure 1A). Furthermore, introducing a single point mutation into the canonical CRE motif (a G-to-A alteration at position −41, mCRE) of the TH promoter caused more than 85% inhibition of the PPA-induced reporter gene activity. We had similar results obtained previously for BA and VPA [60], [58], suggesting involvement of Ca^2+/^cAMP-mediated signaling pathways in the transcriptional effects of structurally related SCFA.

To test this possibility, we also evaluated the role of the general transcription factor CREB in PPA-induced activation of TH gene promoter. In these experiments, PC12 cells were transiently transfected with plasmids carrying the luciferase reporter gene under the control of rat TH promoter in combination with either wild type CREB (positive control); dominant negative CREB expression vector(s) or unrelated DNA (pCMV-CAT expression vector) as we described before [56]. PPA was given one day before and immediately after the transfection. Over-expression of non-functional CREB mutants unable to bind to their cognate DNA enhancer (K-CREB) or to get transactivated in response to activation of different signaling cascades (133CREB) significantly reduced the PPA-triggered increases in reporter gene activity (Fig. 1B). These results indicate that CREB or a related protein(s) and signaling pathways converging on them are involved in mediating the transcriptional effects of PPA on the TH gene promoter.

### 2. PPA can alter steady state TH mRNA and TH protein levels

**Figure 2:**
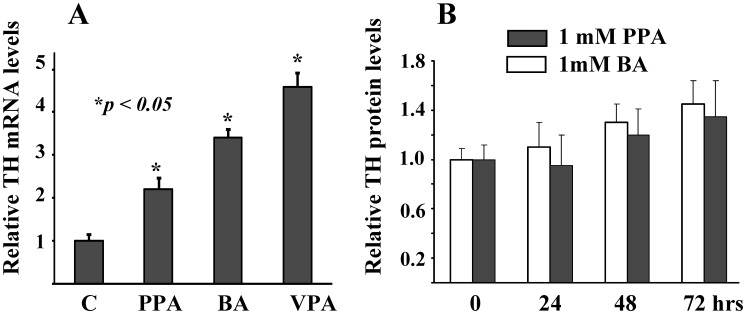
PPA induces accumulation of TH mRNA and TH protein in vitro. PC12 cells were treated with SCFA: propionic acid (PPA, 1 mM), butyric acid (BA, 1 mM) or valproic acid (VPA, 0.1 mM). At indicated times (48 hrs) total RNA or total cell lysates (0, 24, 48 and 72 hrs) were prepared and subjected to Northern (A) or Western blot analyses (B, PPA and BA). The data on Fig. 2A are presented as mean ± SEM value relative to the mRNA levels in control, vehicle treated cells. 18S rRNA was used as a house keeping gene for normalization of data. *P<0.05 SCFA vs. vehicle treated. For Western blotting (2B) cells were lysed after indicated incubation times and equal amounts of proteins were subjected to analyses using TH-specific antibody as described in Experimental procedures. Membranes were re-probed with β-actin specific antibody as loading control. The autoradiographs were analyzed by densitometry. Values are presented as the ratio of TH to that of beta-actin as fold difference from control, vehicle- treated cells. N = 6–8 independent samples per group, and the experiment was repeated twice with similar results [63].

Our previous data revealed that exposure of PC12 cells to low concentrations of butyrate resulted in accumulation of endogenous TH mRNA (see [64], [63]). To compare the effect of structurally-related SCFA on TH mRNA levels, PC12 cells were treated 1 mM BA, 1 mM PPA or 0.1 mM VPA for 48 hours. All treatments resulted in a statistically significant increase in TH mRNA levels (Fig. 2A).

To determine whether SCFA-induced alterations in steady state TH mRNA levels were reflected in changes in TH protein, we treated PC12 cells with 1 mM PPA or BA for 24, 48 and 72 hours. Controls received only vehicle. Exposure to SCFA resulted in gradual increases in the relative immunoreactive TH protein levels, suggesting that the capacity of the cells to produce catecholamines is elevated (Figure 2B).

### 3. Structurally related SCFA regulate similar genetic networks in PC12 cell model

To identify other potential cellular targets for SCFAs, we used microarray technology allowing genome-wide simultaneous measuring of changes in gene expression. PC12 cells were exposed to SCFA (1 mM BA, 6 mM BA, 1 mM PPA and 10 mM PPA), or vehicle for 48 hours. Total RNA was isolated and subjected to microarray analysis using Affymetrix GeneChip Rat Genome 230 2.0 microarray as described in Methods. Data was ranked according to significant up-regulation or down-regulation of twofold or more in SCFA-treated groups compared to their respective control groups (*P<0.05).

Only few affected genes (79) fit the criteria when cells were treated with low dose of 1 mM PPA. The expression of a significantly higher number of genes was altered following exposure to 10 mM PPA (1587). At both doses, BA was more effective than PPA as it was evident by the number of differentially expressed transcripts and correlated with the higher potency of BA to induce histone hyperacetylation and alter global gene expression. For example, a total of 421 genes were significantly altered following treatment with 1 mM BA, and 3148 for 6 mM BA group. We report here the results from MetaCore enrichment analysis performed for 10 mM PA and 6 mM BA groups.

**Figure 3:**
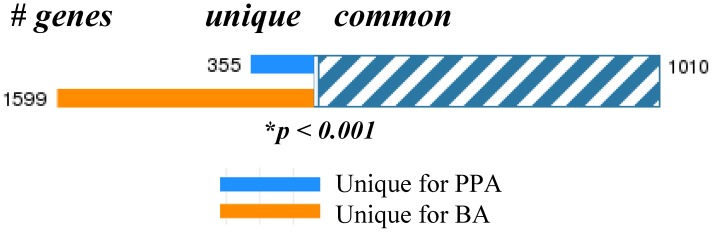
Differential gene expression profiles evoked by SCFA in PC12 cells. Total RNA was isolated from PC12 cells following 48 hrs exposures to BA (6 mM), PPA (10 mM) or vehicle and subjected to microarray analysis. Genes that changed two-fold or greater in SCFA-treated groups (*p<0.05) compared to the control (vehicle treated PC12 cells) group were imported into MetaCore GeneGo for comparison analyses. Differentially expressed unique (1599 for BA and 355 for PPA experimental group) and common for both groups genes (1010) were identified. Fisher's exact test p-value was <2.2e-16.

Comparison analysis revealed 1599 differentially expressed genes unique for the BA experimental group and 355 for the PPA group. A large number of genes (1010) were identified as common for both, BA and PPA groups (Fig. 3, complete lists provided in Table S1, S2, S3). Examples of the highest magnitude affected common genes are given on Table 2. In most cases the extent of induction/reduction of gene expression triggered by BA was more pronounced compared to PPA with the exception of only 16 genes (Table 3). There was also a small subset of genes (21) that change in opposite direction for PPA and BA (Table 4).

**Table 2:** Comparison analysis of differentially expressed genes triggered by PPA and BA in PC12 cells: Top 10 most affected common genes.

Symbol	Gene	PPA	BA
**COL5A3**	Collagen Alpha3 (V) chain	↑644.5	↑1048.6
**COX6B2**	Cytochrome c oxidase subunit VIb	↑6.634	↑156
**CA5B**	Carbonic anhydrase 5B, mitochondrial	↑8.381	↑91.615
**CD40**	Tumor necrosis factor receptor superfamily - member 5	↑33.828	↑90.103
**ICAM1**	Intercellular adhesion molecule 1	↑3.287	↑89.463
**HPD**	4-hydroxyphenylpyruvate dioxygenase	↑2.821	↑82.462
**PPP1R1B**	Protein phosphatase 1 regulatory subunit 1B	↑2.158	↑54.368
**SH3KBP1**	SH3 domain-containing kinase binding protein 1	↑2.8	↑40.719
**SELENBP1**	Selenium-binding protein1	↑14.577	↑25.455
**SPHK1**	Sphingosine kinase 1	↑5.177	↑19.341
**TTK**	Dual specificity protein kinase TTK	↓92.8	↓189.1
**TOP2A**	DNA topoisomerase 2 alpha	↓16.8	↓178.7
**NDC80**	Kinetochore protein HEC1	↓54.1	↓170.2
**RRM2**	Ribonucleoside –diphosphate reductase	↓12.9	↓160.9
**ALDOA**	Fructose-bisphosphate aldolase A	↓22.88	↓142.7
**CCDC95**	Coiled coil domain containing protein 95	↓22.8	↓142.7
**KIAA0101**	PCNA-associated factor	↓22.88	↓142.7
**GTSE1**	G2 and S-phase expressed protein 1	↓7.6	↓122.9
**MKI67**	Antigen KI-67	↓16.631	↓85.181
**ECT2**	Protein ECT2	↓15.863	↓82

The symbols ↑ and ↓ indicate up- and down-regulated genes, respectively. Numbers following the ↑ or ↓ symbols represent the fold-change for the gene expression level. Sorting was done according to the magnitude of the signal (fold change) in BA group. The top 10 for each, upregulated and down regulated genes are shown. For more details, see Table S3.

**Table 3:** Examples of common genes which were affected more by PPA.

Symbol	Gene	PPA	BA
**CD7**	T-cell antigen CD7	↑33	↑18.3
**FBP2**	Fructose-1,6-biphosphatase isozyme 2	↑27.96	↑4.18
**STYXLI**	Serine/threonine/tyrosine-interacting like protein 1	↑12.9	↑9.2
**DYSF**	Dysferlin	↑12.7	↑4.1
**CBS**	Cystathionine beta-synthase	↑10.6	↑2.25
**TPPP3**	Tubulin polymerization-promoting protein 3	↑6.82	↑4.2
**DRD1IP**	D1dopamine receptor-interacting protein calcyon	↑6.1	↑3.1
**SEMA6A**	Semaphorin 6A	↑6.1	↑2.7
**CGN**	Cingulin	↑5.98	↑2.26
**TUFT1**	Tuftelin	↑5.98	↑2.2
**FOS**	Proto oncogene protein c-fos	↑5.86	↑2.2
**TCEA3**	Transcription elongation factor A protein 3	↑5.82	↑3.77
**RKAG2**	5′-AMP-activated protein kinase gamma 2	↓13.6	↓2.1
**BARD1**	BRCA1-associated RING domain protein	↓7.8	↓4.4
**CCNE2**	G1/S specific cyclin-E2	↓4.48	↓2.8
**SYCP3**	Synaptonemal complex protein 3	↓3.98	↓2.2

Sorting was done according the magnitude of the signal (most affected, fold change) in PPA group for both upregulated (↑) and down regulated genes (↓).

**Table 4:** Common genes oppositionally affected by PPA and BA.

Symbol	Gene	PPA	BA
**SPCS3**	Signal peptidase complex subunit 3	↓2.9	↑307.5
**GAS6**	Growth arrest specific protein 6	↓6.6	↑20.48
**RGS2**	Regulator of G-protein signaling 2	↓2.4	↑10.4
**HLA-G**	HLA class 1 histocompatibility antigen alpha G	↓2.4	↑6.71
**NCAM1**	Neural cell adhesion molecule 1	↓3.0	↑ 4.4
**HABP4**	Intracellular hyaluronan binding protein 4	↓2.2	↑4.03
**BRD2**	Bromodomain- containing protein 2	↓2.5	↑3.4
**RGS3**	Regulator of G-protein signaling 3	↓2.1	↑2.9
**NUBP1**	Nucleotide-binding protein 1	↓2.2	↑2.69
**SLIT3**	Slit homolog 3	↑3.3	↓21.3
**LOXL3**	Lysyl oxidase homolog 3	↑2.73	↓5.7
**CAMPI**	Secretory carrier- associate membrane protein 1	↑3.8	↓3.98
**SEPT8**	Septin 8	↑2.48	↓3.9
**PPP2R4**	Serine/threonine phosphatase 2A, subunit B	↑2.44	↓3.5
**IRS2**	Insulin receptor substrate 2	↑2.0	↓3.47
**RAPGEF5**	Rap guanine nucleotide exchange factor 5	↑2.01	↓3.3
**ZNFX1**	NFX1-type zinc finger containing protein 1	↑2.59	↓3.06
**JPH3**	Junctophilin	↑2.0	↓2.6
**TCF4**	Transcription factor 4	↑2.04	↓2.31
**IPO9**	Importin 9	↑2.09	↓2.12
**WNK2**	Serine/threonine protein kinase WNK2	↑3.6	↓2.1

Sorting was done by the magnitude of the signal in BA group for both, upregulated (↑) and down regulated (↓) genes.

### 4. Specifically Affected Gene Systems

Differentially expressed common genes included but were not limited to the following: those involved in the biosynthesis and degradation of dopamine, norepinephrine and serotonin (i.e. TH, DBH, DDC, TPH, GTPCH, COMT, MAOA), mitochondrial/fatty acid metabolism (i.e. 3-ketoacyl-CoA thiolase, ACAA2; acetyl CoA acetyltransferaseACAT2; long chain fatty acid CoA ligase 5- ACSL5; 3-hydroxybutyrate dehydrogenase type 2, BDH2; Glycerol-3-phosphate dehydrogenase, GPD1; hormone sensitive lipase, LIPE), oxidative stress (GPX3-glutathione peroxidase; APOE- apolipoprotein E; MT3- metallothionein-3; DUSP1 dual specificity protein phosphatase 1), gap junction proteins, signaling pathways (cAMP/Ca2+, MAP kinase) receptors (nicotinic, GABA), see Tables 5–8. The complete list of commonly affected by SCFA genes is available in Table S3. For a few selected genes, the expression patterns were validated by Northern blot analyses (Table 1).

**Table 5:** Gene ontology annotation analysis: Oxidative stress.

Symbol	Gene	PPA	BA
**GPX3**	Glutathione Peroxidase, functions in the detoxification of hydrogen peroxide	↑5.797	↑7.459
**CYGB**	Cytoglobin, encodes a globin protein found in vertebrate cells		↓4.574
**APOE**	Apolipoprotein E precursor, essential for the normal catabolism of triglyceride-rich lipoprotein constituents		↑2.28
**MT3**	Metallothionein-3, growth inhibitory factor	↑11.302	↑8.069
**BNIP3**	BCL2/adenovirus E1B 19 kDa protein-interacting protein 3, protects from virally-induced cell death and apoptosis		↓2.195
**EPHX2**	Epoxide hydrolase 2, binds to specific epoxides and converts them to the corresponding dihydrodiols	↑2.807	↑4.989
**CYGB**	Cytoglobin		↓4.574
**DUSP1**	Dual specificity protein phosphatase 1, induced by oxidative/heat stress and growth factors, may play a role in negativeregulation of cell proliferation	↓2.068	↓2.736
**FOXM1**	Forkhead box protein M1, encodes transcriptional activator involved in cell proliferation	↓5.286	↓7.152
**NQO1**	NAD(P)H dehydrogenase [quinone] 1, encodes cytoplasmic 2-electron reductase	↓3.195	↓2.749

Total gene IDs in the ontology- 106, matches for PPA – 9, for BA - 12.

**Table 6:** Gene ontology annotation analysis: Dopamine & serotonin pathways.

Symbol	Gene	PPA	BA
**COMT**	Catechol O-methyltransferase catalyzes the transfer of a methyl group from S-adenosylmethionine to catecholamines	↓2.693	↓2.742
**TH**	Tyrosine 3-monooxygenase, rate limiting enzyme in the synthesis of catecholamines, converts tyrosine to dopamine	↓<2	↓7.069
**DBH**	Dopamine beta-hydroxylase, oxidoreductase which Converts dopamine to norepinephrine	↓2.157	↓17.434
**DDC**	Aromatic-L-amino-acid decarboxylase catalyzes the decarboxylation of L-3,4-dihydroxyphenylalanine to dopamine, L-5-hydroxytryptophan to serotonin, L-tryptophan to tryptamine.	↓3.181	
**MAOA**	Amine oxidase [flavin-containing] A catalyzes the oxidative deamination of dopamine, norepinephrine, and serotonin	↑2.049	↑2.323
**GTPCH**	GTP cyclohydrolase I, first and rate limiting enzyme in tetrahydrobiopterin biosynthesis, an essential cofactor for aromatic amino acid hydroxylases and nitric oxide synthases	↑2.671	
**TPH1**	Tryptophan 5-hydroxylase 1 catalyzes the first rate limiting step in the biosynthesis of serotonin	↑2.869	
**SLC6A2**	Sodium-dependent noradrenaline transporter		↓8.594
**ADCY2**	Adenylate cyclase type 2, catalyzes the formation of second messenger cAMP		↑2.255
**ADCY3**	Adenylate cyclase type 3		↓2.651
**CASP3**	Caspase-3 precursor, plays a role in the execution-phase of cell apoptosis		↑2.942
**DUSP1**	Dual specificity protein phosphatase 1	↓2.068	↓2.736
**FOS**	Proto-oncogene protein c-fos encodes leucine zipper proteins that dimerize with JUN family proteins forming the AP-1 transcription factor complex	↑5.866	↑2.22
**PPP1R1B**	Protein phosphatase 1 regulatory subunit 1B, encodes a bifunctional signal transduction molecule, kinase or phosphatase inhibitor	↑2.158	↑54.368
**CYP2D6**	Cytochrome P450 2D6 encodes a member of the cytochrome P450 super family of monooxygenases	↑2.283	↑6.308
**APP**	Amyloid beta A4 protein precursor encodes a cell surface receptor and transmembrane precursor protein cleaved by secretases to form a number of peptides	↓3.049	↓5.745
**ARRB2**	Beta-arrestin-2, thought to participate in agonist-mediated desensitization of G-protein-coupled receptors and to cause specific dampening of cellular responses to stimuli		↑2.071
**SLC18A1**	Vesicular monoamine transporter (dopamine gene target)	↓6.834	↓82.613

Total gene IDs in the ontology – 85, matches for PPA – 13, for BA -15.

**Table 7:** Gene ontology annotation analysis: Fatty acid metabolism.

Symbol	Gene	PPA	BA
**ACAA2**	3-ketoacyl-CoA thiolase, mitochondrial catalyzes the last step of the mitochondrial fatty acid beta-oxidation	↑2.608	↑2.982
**ACAT1**	Acetyl-CoA acetyltransferase, mitochondrial, catalyzes the reversible formation of acetoacetyl-CoA from two molecules of acetyl-CoA	↑2.003	
**ACAT2**	Acetyl-CoA acetyltransferase, cytosolic encodes enzyme involved in lipid metabolism, acetoacetyl-CoA thiolase	↓4.148	↓3.199
**ACADSB**	Short/branched chain specific acyl-CoA dehydrogenase, mitochondrial precursor, catalyzes the dehydrogenation of acyl-CoA derivatives in the metabolism of fatty acids or branch chained amino acids		↑2.776
**ACADVL**	Very long-chain specific acyl-CoA dehydrogenase, mitochondrial precursor, catalyzes the first step of the mitochondrial fatty acid beta-oxidation pathway		↑2.251
**ACSL5**	Long-chain-fatty-acid-CoA ligase 5, converts free long-chain fatty acids into fatty acyl-CoA esters	↓3.046	↓4.13
**ACSL6**	Long-chain-fatty-acid-CoA ligase 6, catalyzes the formation of acyl-CoA from fatty acids, ATP, and CoA		↓2.283
**ACOT7**	Cytosolic acyl coenzyme A thioester hydrolase, hydrolyzes the CoA thioester of palmitoyl-CoA and other long-chain fatty acids		↓2.274
**CRAT**	Carnitine O-acetyltransferase, catalyzes the reversible transfer of acyl groups from an acyl-CoA thioester to carnitine and regulates the ratio of acylCoA/CoA in the subcellular compartments		↓2.777
**DECR**	12,4-dienoyl-CoA reductase, mitochondrial, accessory enzyme in beta-oxidation and metabolism of unsaturated fatty enoyl-CoA esters	↑3.7	↑3.537
**MUT**	Methylmalonyl-CoA mutase, mitochondrial, vitamin B12-dependent enzyme which catalyzes the isomerization of methylmalonyl-CoA to succinyl-CoA		↓2.692
**FABP3**	Fatty acid-binding protein, heart, thought to participate in the uptake, intracellular metabolism and/or transport of long-chain fatty acids		↓3.447
**SLC27A1**	Long-chain fatty acid transport protein 1		↑2.537
**PRKAA1**	5′-AMP-activated protein kinase alpha-1, the catalytic subunit of the 5′-prime-AMP-activated protein kinase (AMPK) a cellular energy sensor		↑2.715
**BDH2**	3-hydroxybutyrate dehydrogenase type 2	↑4.612	↑53.067
**GPD1**	Glycerol-3-phosphate dehydrogenase [NAD+], plays a critical role in carbohydrate and lipid metabolism	↓2.442	↓9.572
**LIPE**	Hormone-sensitive lipase	↑2.238	↑2.575
**LPL**	Lipoprotein lipase precursor, has dual functions of triglyceride hydrolase and ligand/bridging factor for receptor-mediated lipoprotein uptake		↓8.57

Total gene IDs in the ontology - 90, matches for PPA – 8, for BA – 18.

**Table 8:** Gene ontology annotation analysis: GAP junction proteins.

Symbol	Gene	PPA	BA
**GJB5**	Gap junction beta-5 protein, involved in intercellular communication related to epidermal differentiation and environmental sensing		↓2.624
**EGFR**	Epidermal growth factor receptor precursor		↓2.688
**PDGFRA**	Alpha-type platelet-derived growth factor receptor		↓2.152
**DBN1**	Drebrin, cytoplasmic actin-binding protein thought to play a role in the process of neuronal growth	↑2.128	
**CTNNB1**	Catenin beta-1, part of a complex of proteins that constitute adherens junctions		↑2.21
**TJP1**	Tight junction protein ZO-1		↑2.687
**PLCB1**	1-phosphatidylinositol-4,5-bisphosphate phosphodiesterase, catalyzes the formation of inositol 1,4,5-trisphosphate and diacylglycerol from phosphatidylinositol 4,5-bisphosphate		↑2.663
**SRC**	Proto-oncogene tyrosine-protein kinase Src, involved in regulation of embryonic development and cell growth	↓2.286	↓5.815
**TUBB2B**	Tubulin beta-2B chain, binds GTP and is a major component of microtubules.	↑3.164	
**TUBB2C**	Tubulin beta-2C chain	↓2.047	
**GRB2**	Growth factor receptor-bound protein 2, binds the epidermal growth factor receptor, involved in signal transduction		↓3.034
**RAF1**	RAF proto-oncogene serine/threonine-protein kinase, encoded protein is a MAP kinase kinase kinase (MAP3K)		↓2.462
**ITPR2**	Inositol 1,4,5-trisphosphate receptor type 2	↑4.719	↑8.015
**PLCB1**	1-phosphatidylinositol-4,5-bisphosphate phosphodiesterase		↑2.663
**PRKCG**	Protein kinase C gamma type, expressed solely in the brain and spinal cord, localization is restricted to neurons		↑2.203
**ADCY2**	Adenylate cyclase type 2		↑2.255
**ADCY3**	Adenylate cyclase type 3		↓2.651

Total gene IDs in the ontology - 101, matches for PPA - 6, for BA- 12.

### 5. Gene Networks

The top five most relevant gene networks, identified using the Analyze Networks (AN) algorithm with default settings (MetaCore, [74]) are listed in Table 9. Processes involved in the cell cycle and regulation of the progression through the cell cycle are the most affected, consistent with the ability of HDACI to induce growth arrest, cell differentiation, and chromatin remodeling in a variety of cell lines [47].

**Table 9:** Most relevant biological networks identified for common genes.

#	Processes	Pathways	P-value	Zscore
**1**	Cell cycle, mitotic cell cycle	230	1.73e-19	15.25
**2**	Cell cycle, regulation of progression through cell cycle	147	1.16e-15	13.07
**3**	Intracellular signaling cascade, localization of the cell, cell motility	71	1.84e-5	5.90
**4**	G-protein signaling coupled to cAMP, regulation of adenylate cyclase	52	4.02e-17	13.97
**5**	Actin filament polarization and/or depolarization, protein polarization	0	2.98e-68	34.11

The list of biological networks was generated using the Analyze Networks (AN) algorithm with default settings (MetaCore), based on relative enrichment with the uploaded data and the relative saturation of networks with canonical pathways.

P-value: represents the probability for a particular mapping of an experiment to a map (or network, or process) to arise by chance considering the number of genes in the experiment versus the number of genes in the map within the “full set” of all genes on maps. Z-score ranks the sub-networks of the AN algorithm with regard to their saturation with genes from the experiment.

**Figure 4:**
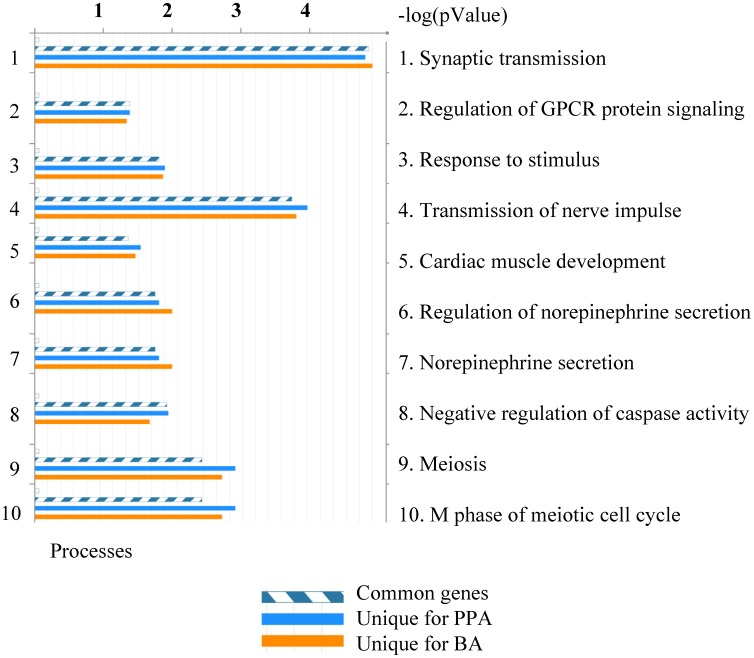
Enrichment analysis of differentially expressed genes: distribution by gene ontology (GO) processes. Differentially expressed genes in BA and PPA groups (t-test compared to control group p<0.01) were subjected to enrichment analysis (which consists of matching gene IDs for the common, similar and unique sets of the uploaded files with gene IDs in functional ontologies in MetaCore). The figure illustrates the distribution by GO processes. The gene content is aligned between all uploaded files. The set of common gene IDs is marked as blue and white stripes. The unique genes for the files are marked as colored bars (BA - orange; PPA- blue). The sorting was done by common gene IDs; p-value was set for 0.05; both signals (induced and repressed) were included. The data shown are for sorting method “similarity by”. The degree of “relevance” to different categories for the uploaded datasets is defined by p values, so that the lower p-value gets higher priority. The top 10 processes are listed based on their −log (p-value).

In order to explore the biological significance of SCFA-mediated gene expression changes, we analyzed their Gene Ontology (GO) classification using MetaCore (Fig. 4). The most highly represented processes (sorting method “similarity by”) include: synaptic transmission, regulation of G-protein coupled receptor signaling, response to stimulus, transmission of nerve impulse, cardiac muscle development, regulation of NE secretion, NE secretion.

## Discussion

Emerging evidence links SCFA from diet, cellular metabolism or pharmacological treatments with regulation of neuronal gene expression, attendant neuronal function and even behavior [34], [35], [36], [37], [38], [45], [39], [41], [42], [27], [43], [44], [64], [58], [63], [75], rev. in [3]. In this study, we demonstrate that PPA can induce TH gene transcription over a wide dose range (0.1 mM to 10 mM) in PC12 cells, similar to the effects of BA [63], [75]. The general transcription factor CREB (or CREB-related proteins) and signaling pathways converging on it are involved in the transcriptional regulation of the TH gene by PPA or BA. At lower concentrations, PPA - triggered changes in TH gene transcription are reflected in accumulation of TH mRNA and TH protein, which are markers of increased catecholamine production. Genome-wide expression profiling following exposure to SCFA enabled us to identify large number of affected genes and networks, common for both PPA and BA, including catecholaminergic pathways and GO processes implicated in ASD.

The short chain fatty acid BA is a well known HDACI shown to induce differentiation, growth arrest and chromatin remodeling in a variety of cell lines, associated with specific-gene transcription regulation properties [47], [48], [49], [50]. The effects of BA are similar to its structurally related branched short chain fatty acid with a known causal role in ASD VPA, [51], [52] which produces an acceptable animal model for the condition [68], [70], [69], [76], [77]. PPA also exhibit HDACI properties in non-neuronal cell lines, although it is less potent than BA [53]. Here using a PC12 cell model we show that PPA activates TH promoter-driven reporter gene expression in a concentration-dependent manner (Fig. 1A), similar to other HDACI like BA, VPA, trichostatin A and phenylbutyrate [75], [58]. This induction was dependent on the binding ability and transactivation status of the general transcription factor CREB - a stimulus induced transcription factor, phosphorylated at Ser133 by diverse array of protein kinases including PKA, Ca2+/CAM kinases and Ras/MAPK pathway [78], [67].

Our data correlate well with the fact that SCFA may enter the cell by passive diffusion and/or active transport via specific monocarboxylic acid/ketone transporters [79], [80] and inhibit HDAC, but they can also interact with membrane receptors and trigger activation of different signaling cascades to alter the expression of specific genes [81]. In this regard, PPA was characterized as the most potent agonist for GPCR41 and GPCR43 which are coupled to inositol 1,4,5-trisphosphate formation, intracellular Ca2+ release, ERK1/2 activation [82], [83]. G-protein signaling coupled to cAMP and regulation of adenylate cyclase was also among the top 5 networks identified for common genes in our microarray data analyses (see Table 9). In rodents and humans, PPA is found to directly regulate GPCR41-mediated sympathetic outflow and thereby to modulate body energy expenditure in maintaining metabolic homeostasis [84], [85].

### PPA modulation of inflammatory processes/lipid homeostasis

PPA can also exert its effect by activating nuclear receptor PPAR gamma [86]. We have demonstrated previously that PPAR gamma receptors are expressed in PC12 cells and are involved in the independent regulation of catecholaminergic and opioid pathways by SCFA and related drugs like 4-phenylbutyrate [75]. Our microarray analyses showed SCFA - altered expression of genes, involved in fatty acid metabolism and transport, ketogenesis and ketone body metabolism, as well as triacylglycerol metabolism (Table 7). These findings are of particular interest since abnormal lipid metabolism has been reported in ASDs and ASD-related disorders [87], [88], [89], [90], [91], [92]. Moreover, clinical studies suggest improvements in core symptoms of patients with ASDs following supplementation with polyunsaturated fatty acids (PUFAs) [93], [94], [95] and we observed altered lipid profiles in rat brain phospholipids following infusion with both PPA and, to a lesser extent, BA [37], [96].

### PPA activation of CREB dependent catecholaminergic pathways

The microarray data reported here are also consistent with the activation of CREB pathway – i.e. increased expression of CREB3L1 was observed in PPA (2.33 fold) and BA (4.2 fold) group, accompanied by elevated MAP4K1 (2-fold and 3.6 fold resp.), CAMK2G (2-fold for both SCFA) and CAMKV (increased by approximately 3-fold by PPA and BA). Increased P-CREB immunoreactivity was observed in ASD- hippocampus and white matter regions following intracerebroventricular infusion of SCFA (ASD rodent model, [35], [3]). Moreover, our prior work with BA is consistent with a signal transduction system involving CREB and converging on the CRE and a novel upstream 5′ regulatory element (BRE; GCCTGG at −509 to −504 of both, the rat TH and proenkephalin promoters [60], [58], [55], [56].

The ability of PPA to activate endogenous TH transcription at low concentrations (1 mM) results in accumulation of TH mRNA and TH protein (Fig. 2) indicative of increased cell capacity to produce catecholamines. This observation is similar to the effects of BA and VPA in PC12 cells we reported before [63], [58]. Furthermore, it provides a possible molecular mechanism to account for the hyperactivation of the mesocortical dopamine system suggested in several clinical reports of ASD [97], [98] and in animal models of ASD [66], [99]. Other 4 carbon structures (i.e. GABA or γ-OH butyrate – which is accumulated as a result of succinic semialdehyde dehydrogenase deficiency, a rare autosomal recessive disorder which presents with autistic behaviors, [100]) and following ketogenic diet administration, a possible therapy for ASD, also have potent effects on CNS function via the dopaminergic system suggesting a broader implication of this putative mechanism.

Although the stimulatory effect of SCFA on the TH promoter is proportional to the drug dose used (Fig. 1A for PPA, also previously shown by us for BA, phenylbutyrate and VPA [75], [58]), TH mRNA and protein fall below basal levels at high concentration [58], [63], which was associated with increased TH mRNA destabilization presumably via a butyrate response factor (BRF) acting at a 3′ untranslated region [58], [61], [63], [101]. These high concentrations are comparable to the doses of brief pulse intracerebroventricular infusions used in our rat ASD model [35] and blood PPA concentrations in human propionic acidemia (4400 uM/L, see [25], [27]). An interesting new finding of the present study is the **bi-directional** perturbation of the dopaminergic pathways by both PPA and BA (Table 1, 6 and Table S3). It should be emphasized that this novel SCFA-triggered mode of regulation is different from the reported multiple studies (rev. in [102], [103], [104], [59]) where diversified stimuli (cyclic AMP analogues, phorbol esters, growth hormones, glucocorticoids, depolarization agents) each increased TH mRNA levels and stimulated TH promoter activity in PC12 cells. Whether a similar bi-directional dysregulation of the brain catecholaminergic system occurs in response to excessive concentrations of SCFA exist *in vivo* remains to be examined. A bi-directonal system is intriguing since high concentrations of SCFA down regulated not only TH, but also DBH, DDC and COMT genes in PC12 cells (our results reported here, also [62]). Loss or down regulation of the DBH gene is strongly associated with deficits in affiliative and self injurious behaviors in several animal models (rev. in [105], [106]) and with ASD phenotype in humans [107], [108], [109], [110].

### Serotonergic/Cholinergic systems

Of interest, only PPA was able to alter the serotonin system in PC12 cells by inducing the expression of Tryptophan 5-hydoxylase 1 (TPH1, rate-limiting enzyme in the synthesis of the neurotransmitter serotonin) and GTP cyclohydrolase 1 (GTPCH - the rate limiting enzyme in BH4 biosynthesis, an essential cofactor of three aromatic amino acid hydrolases including TPH and TH); Table 6. Dysfunctional serotonin signaling has been suggested as an etiologic factor in ASD (rev. in [111]). Serotonin is linked to the mediation of several psychological processes, many of which are altered in ASD patients, including mood, social interaction, sleep, obsessive compulsive behaviors and aggression. Similar symptoms were also observed in our PPA animal model of ASD [3], [44].

During the past few years, studies have begun to suggest that cholinergic systems in the brain might also be implicated in ASD [112], [113]. We also found altered expression of CHRNA3, CHRNB1 (both down regulated by BA), CHRNA5 and CHRNB4 (up-regulated by PPA 3.2 and 5-fold, see Tables S1 and S2, and GSE56516).

### Mitochondrial dysfunction/oxidative stress

The brain is vulnerable to mitochondrial dysfunction, oxidative stress and excitotoxicity [114]. Consistent with the effects of SCFA (PPA and/or BA) on cellular metabolism [35], [37], [96], [115], the data presented in Table 5 are similar to the clinical presentation of patients with ASD who show metabolic dysfunction, including impairments in B12, glutathione, or carnitine metabolism [116], [117], [118], [43] and mitochondrial disorder/dysfunction [119], [120]. VPA similarly alters mitochondrial metabolism and causes depletion of carnitine stores and encephalopathy [121], [122]. Of note, we have shown specific alterations of carnitine and mitochondrial metabolism in a large cohort of ASD patients predicted with the PPA rodent model suggesting mitochondrial dysfunction, which also alters gene expression [123], may play a key role in ASD pathogenesis [45], [3], [4].

### General effects of SCFA

DNA microarray technology enables simultaneous detection of perturbations in thousands of genes in a single experiment. The present study also identified a large number of affected genes following exposure of PC12 cells to high concentrations of SCFA using a criteria of ≥2-fold change and a *P*≤0.05 (Figure 3, Tables S1, S2, S3): 1599 unique for BA, 355 unique for PPA and 1010 common for both SCFA. Upon closer look at the expression profiles triggered by PPA and BA, we found a set of 16 common genes altered to a higher extent after PPA treatment (Table 3) and a total of 21 common genes affected in the opposite direction by PPA and BA (Table 4). Together, these data confirm the notion that in addition to the common ability to cause HDAC inhibition, other processes in PC12 cells are influenced in a PPA-and/or BA specific manner providing further layers of regulation and potential for neuronal plasticity.

### Alteration of ASD associated genes FMR1, Neurexins, Neuroligins

In addition, we identified a number of genes (listed in Table 10) that were previously implicated in ASD or candidate genes for ASD from evaluation of copy number variation genetic studies. For example, loss of function of Fragile X Mental Retardation 1 gene (FMR1) causes Fragile X syndrome with up to 90% of affected children exhibiting ASD symptoms [124], [125], [126]. Treatment of PC12 cells with PPA or BA caused down regulation of FMR1 gene expression (Table 10). Neurexin 1 (NRXN1) is another gene considered to be causal for ASD [127], [128] which was also down regulated following BA administration. Many genes associated with ASD are also involved in the neuroligin-neurexin interaction at the glutamate synapse [129], [28].

**Table 10:** SCFA alter the expression of genes implicated in ASD in human and animal studies.

*Genes implicated in ASD (Copy number variations)*			
Symbol	Gene	PPA	BA
**C3orf58**	Uncharacterized protein C3orf58, renamed deleted in autism 1, encodes signal peptide for targeting to the secretory pathway		↑4.539
**CACNA1C**	Voltage-dependent L-type calcium channel alpha-1C mediate the influx of calcium ions into the cell upon membrane polarization		↓2.093
**DMPK**	Disks large-associated protein 2, dystrophy myotonica protein kinase	↑2.258	↑3.018
**FMR1**	Fragile X mental retardation 1 protein, negative regulator of translation, plays key role in neuroplasticity	↓2.289	↓2.383
**NIPBL**	Nipped-B-like protein, facilitates long distance enhancer-promoter communications	↓2.766	
**NLGN2**	Neuroligin-2 , post synaptic cell adhesion molecule of inhibitory synapses		↓2.471
**NRXN1**	Neurexin-1-alpha, 1-beta, pre-synaptic protein that forms complexes with neuroligins or other partners to shape synaptic neurotransmission	↓2.227	
**PTEN**	Phosphatidylinositol-3,4,5-trisphosphate 3-hosphatase and dual- specificity protein phosphatase, tumor suppressor negatively regulate AKT/PKB signaling		↑2.922
**RAI1**	Retinoic acid-induced protein 1, transcription factor induced by retinoic acid		↓2.566

*ASD Candidate genes:*			
**ASTN1**	Astrotactin-1 precursor, neuronal adhesión molecule, involved in glia-guided neuronal migration		↓2.68
**DLGAP2**	Disks large-associated protein 2, neuronal postsynaptic density protein	↑2.258	↑3.018
**DPP6**	Dipeptidyl aminopeptidase-like protein 6 auxiliary subunit of Kv4-mediated A-type K(+) channels	↓2.455	↓15.024
**DPYD**	Dihydropyrimidine dehydrogenase [NADP+], involved in uracil and thymidine catabolism		↑5.115
**GABRB1**	Gamma-aminobutyric acid receptor subunit beta-1		↑2.368
K**CNMA1**	Calcium-activated potassium channel alpha-1 the channels control neuronal excitability and smooth muscle tone	↓2.885	
**MAOA**	Monoamine oxidase [flavin-containing] A degrades amine neurotransmitters	↑2.049	↑2.323
**MTF1**	Metal regulatory transcription factor 1, Zn binding transcription factor that mediates regulation of metallothionein genes		↑2.056
**PCDH9**	Protocadherin-9, encodes a cadherin-related neuronal receptor that localizes to synaptic junctions		↑22.029
**PCDH10**	Protocadherin alpha 10, cadherin-related neuronal receptor believed to play a role in establishment and function of specific cell-cell connections in the brain		↓2.477
**PIP5K1B**	Phosphatidylinositol-4-phosphate 5-kinase 1 beta	↑2.328	
**PRKAG2**	5′-AMP-activated protein kinase gamma-2, energy-sensing enzyme, composed of a catalytic alpha, noncatalytic beta and noncatalytic regulatory gamma subunit.	↓13.619	↓2.154

Thus, it appears that number PPA and BA affected genes are related to ASD. In other words, the apparent epigenetic regulation of gene expression by gut-microbiota products (SCFA) can in principle result in similar “loss” or “gain” of function and disrupt similar pathways/networks as genetic alterations associated with ASD.

We applied MetaCore Gene Ontology (GO) bioinformatics tool [130] for functional enrichment profiling of our genome-wide differential expression data in response to SCFA (Figure 4). Of note, the top 10 enriched GO terms included synaptic transmission, regulation of G-protein coupled signaling, transmission of nerve impulses. Our data are concordant with the comprehensive review and analysis of published literature and data of ASD genetics [131] including a total of 2193 genes, 2806 SNPs/VNTRs, 4544 CNVs and 158 linkage regions.

In summary, the gene expression analysis in this study provides valuable insight into the complexity of possibilities and specific molecular pathways or processes altered in PC12 cells upon stimulation with SCFA, many of which, directly or indirectly, have been implicated in ASD. The study also underscores a novel capacity of SCFA to regulate multiple pathways and networks, which are relevant to brain functions and behavior. We believe that our data represent a useful initial cell model resource for identifying and functionally testing SCFA-responsive neuronal genes.

Although the preliminary data are compelling, it is important to note that the effects of SCFAs on cellular systems are complex, often indirect, and often time, tissue- and dose-related [8], [3], [132]. In addition to direct HDACI effects, other SCFA effects on GPCR, second messenger/calcium signaling, and mitochondrial/oxidative stress also alter gene expression. One should be cautious in the interpretation of the data from this *in vitro* system, as the effects of PPA on non-neural CNS cells (glia, [133]) neuronal subtypes or systemic effects [134] have not been addressed. At this stage, we do not know the stability of these epigenetic, transcriptional and mRNA destabilization changes. Ongoing research include using whole animal models at specific time periods and routes of PPA exposure to examine the effects of gut derived bacterial metabolites on brain function and behavior, and their possible relation to neurodevelopmental disorders.

We speculate that altered levels of environmental factors (such as SCFA) normally produced in the gut by microbial fermentation of dietary carbohydrates, may:

1. Represent a novel evolutionary link to neural plasticity associated with different behavioral responses to a changing environment.
2. Affect brain and behavior by multiple molecular mechanisms, some of which involve Ca2+/cAMP/CREB signaling pathways.
3. Modulate expression of key genes (neurotransmitters, cell adhesion molecules, genes involved in lipid metabolism, oxidative stress and mitochondrial function) many of which have been implicated in neurodevelopmental disorders, such as ASD.

## Supporting Information

Table S1 List of differentially expressed genes unique for PPA.
(XLS)

Table S2 List of differentially expressed genes unique for BA.
(XLS)

Table S3 List for differentially expressed genes, common for PPA and BA.
(XLS)
